# Mechanism of Arsenic Partitioning During Sulfidation of As-Sorbed Ferrihydrite Nanoparticles

**DOI:** 10.1021/acsearthspacechem.1c00373

**Published:** 2022-07-06

**Authors:** Naresh Kumar, Vincent Noël, Johannes Besold, Britta Planer-Friedrich, Kristin Boye, Scott Fendorf, Gordon E. Brown

**Affiliations:** † Department of Geological Sciences, School of Earth, Energy & Environmental Sciences, Stanford University, Stanford, California 94305-2115, United States; ‡ Center for Environmental Implications of NanoTechnology (CEINT), Duke University, Durham, North Carolina 27708, United States; § Soil Chemistry and Chemical Soil Quality Group, Wageningen University, 6708 PB Wageningen, The Netherlands; ∥ Stanford Synchrotron Radiation Lightsource (SSRL), SLAC National Accelerator Laboratory, 2575 Sand Hill Road, Menlo Park, California 94025, United States; ⊥ Environmental Geochemistry, Bayreuth Center for Ecology and Environmental Research (BayCEER), University of Bayreuth, D-95440 Bayreuth, Germany; # Department of Earth System Sciences, School of Earth, Energy & Environmental Sciences, Stanford University, Stanford, California 94305, United States

**Keywords:** thioarsenic, trithioarsenate, sulfidation, arsenic speciation, mobility

## Abstract

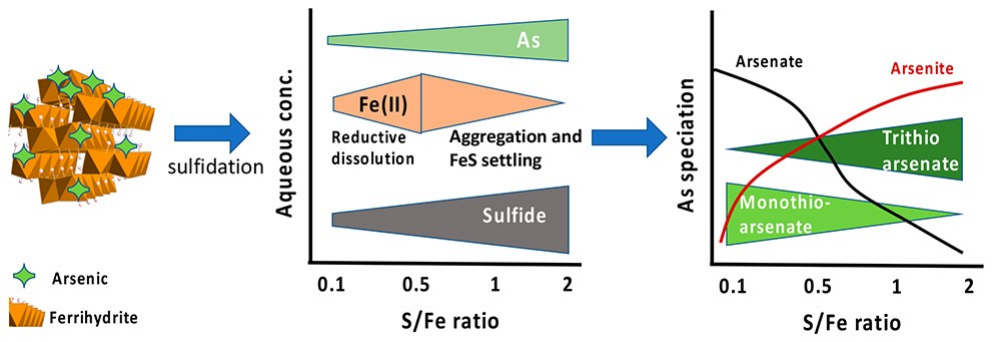

Knowledge of how arsenic (As) partitions among various phases in Fe-rich sulfidic environments is critical for understanding the fate and mobility of As in such environments. We studied the reaction of arsenite and arsenate sorbed on ferrihydrite nanoparticle surfaces with dissolved sulfide at varying S/Fe ratios (0.1–2.0) to understand the fate and transformation mechanism of As during sulfidation of ferrihydrite. By using aqueous As speciation analysis by IC-ICP-MS and solid-phase As speciation analysis by synchrotron-based X-ray absorption spectroscopy (XAS), we were able to discern the mechanism and pathways of As partitioning and thio-arsenic species formation. Our results provide a mechanistic understanding of the fate and transformation of arsenic during the codiagenesis of As, Fe, and S in reducing environments. Our aqueous-phase As speciation data, combined with solid-phase speciation data, indicate that sulfidation of As-sorbed ferrihydrite nanoparticles results in their transformation to trithioarsenate and arsenite, independent of the initial arsenic species used. The nature and extent of transformation and the thioarsenate species formed were controlled by S/Fe ratios in our experiments. However, arsenate was reduced to arsenite before transformation to trithioarsenate.

## 1 Introduction

Arsenic (As) is a highly toxic metalloid and a contaminant of worldwide concern for drinking and irrigation waters. Elevated As concentrations in ground and surface waters result from both natural and anthropogenic sources, and its mobility and toxicity are directly related to the chemical speciation.^1−3^ The fate of As in the environment is inherently linked to the biogeochemistry of iron (Fe)-bearing minerals *via* sorption and (co)-precipitation reactions. Fe(III)-(oxyhydr)oxides are strong sorbents of As and are ubiquitous in soils and near-surface sediments. As a consequence, they exert a dominant influence on the transport, fate, and bioavailability of As.^4,5^ For example, As concentrations as high as 14% by mass have been observed in natural ferrihydrite (∼Fe(OH)_3_) samples.^6^

The reductive dissolution of As-sorbed Fe(III)-(oxyhydr)oxides mediated by microbial Fe or sulfate reduction is believed to be the main cause of elevated concentrations of As in the aqueous phase under anaerobic conditions.^4,7−13^ In reducing environments where dissolved sulfate concentrations are significant, microbial sulfate reduction drives dissolved sulfide production.^14^ Dissolved sulfide is a strong reductant and readily reacts with Fe(III)-(oxyhydr)oxides to produce Fe(II), and during this process, dissolved sulfide oxidizes to elemental sulfur (S^0^).^15−18^ Recent studies confirm that this reaction is primarily controlled by the S/Fe ratio.^18,19^ Kumar et al.^18^ showed that, during sulfidation of Fe(III)-(oxyhydr)oxides at S/Fe ratios ≤0.5, Fe(II) is released to aqueous phase (<0.22 μm) without significant formation and settling of FeS aggregates, but, at S/Fe ratios >0.5, Fe(II) was not detected in the aqueous phase (<0.22 μm), and significant formation and settling of FeS aggregates occurred. However, Noël et al.^19,20^ later confirmed that Fe(II) measured at S/Fe ratios ≤0.5 is present mostly as FeS nanoclusters and not as dissolved Fe(II). It is reasonable to assume that the sulfidation of Fe(III)-(oxyhydr)oxides, owing to reductive dissolution, would have implications for the transformation and/or mobility of any surface-sorbed metals and metal(loid)s, including As. Thus, the fate and behavior of surface-sorbed As would be directly impacted by the S/Fe ratio and, depending on the S/Fe ratio, could have different transformation reaction mechanism(s). For example, at lower S/Fe ratios (≤0.5), As could be released into solution along with the reductive dissolution of Fe(III)-(oxyhydr)oxide surface or can sorb on or coprecipitate with secondary Fe-bearing minerals. In addition, As could also react with S^0^ to form thiolated-As species like monothioarsenate, and at higher S/Fe ratios (>0.5), As could interact with dissolved sulfide to reduce arsenate to arsenite.^13^ We hypothesize that the exact As reaction mechanism will depend primarily on the S/Fe ratio available. However, in natural systems, it is difficult to control or determine the exact S/Fe ratio. Sulfate-reducing microbes could also directly reduce Fe, which would result in the additional release of Fe(II) compared with that from abiotic reduction by dissolved sulfide alone.^17^

Although arsenite generally dominates As speciation in reducing environments, there are mounting evidences that thiolated As species can also (co)exist in sulfidic environments.^12,13,21−26^ Thioarsenates are structural analogues to arsenate that generally form under sulfate-reducing conditions from arsenite by OH^–^/SH^–^ ligand exchange and oxidative addition of S^0^. Thioarsenates are interesting as they contain arsenic in its oxidized state and sulfur in its reduced state and thus could potentially serve as both an electron acceptor and an electron donor.^27^ Although the chemical properties of thiolated As species are still largely unknown, they clearly exhibit sorption properties that are distinct from those of the more widely studied arsenate and arsenite oxoanions, impacting As mobility.^28^ In sulfidic environments, depending on the Fe availability, some free sulfide can become available to complex As, allowing possible precipitation of As-sulfide minerals, such as realgar (α-As_4_S_4_) and orpiment (As_2_S_3_), and/or the formation of thiolated As species.^13,26,29^ However, the geochemical boundaries for the formation of thiolated As species in Fe-rich environments have not been systematically investigated. Our current knowledge of the geochemistry of Fe and As during sulfidation is largely based on field observations and soil-sediment incubation experiments.^12,30,31^ Natural complexity and concurrent competitive processes in natural environments make it difficult to quantify specific sulfide-induced reaction mechanisms and pathways for Fe, S, and As. Although some progress has been made, As speciation in sulfidic environments and the geochemical boundaries of thio-As species in the presence of Fe still remain largely unresolved.

To gain a better understanding of the impact of sulfidation of Fe(III)-(oxyhydr)oxides on the molecular-level speciation and mobility of sorbed As, we have carried out a targeted batch study of abiotic reductive dissolution of arsenite and arsenate sorbed ferrihydrite nanoparticles by dissolved sulfide at various S/Fe ratios (0.1–2.0) at pH 5. Our primary objective was to evaluate the importance of S/Fe ratios in geochemical partitioning of As–S–Fe to understand fate, mobility, and speciation interplay of As in these complex biogeochemical environments. We analyzed As-speciation in the aqueous phase using ion-chromatography coupled with inductively coupled plasma mass spectrometry (IC–ICP-MS) and solid-phase As-speciation using synchrotron-based X-ray absorption spectroscopy (XAS) to develop a more complete reaction mechanism for these systems. This study provides new insights about reaction mechanisms and the fate of arsenic in sulfidic environments and the role of S/Fe ratio in thioarsenate species formation and distribution in reducing systems.

## 2 Materials and Methods

Unless otherwise specified, all experiments were carried out under O_2_-free conditions using O_2_-free Milli-Q water and an anoxic chamber (96% N_2_ + 4% H_2_) equipped with an O_2_ detector and a Pd catalyst (Coy Laboratories). The O_2_-free water used throughout this study was prepared by bringing the Milli-Q water to boil and sparging it with high purity N_2_ gas while cooling to room temperature (∼4 h). No unexpected or unusually high safety hazards were encountered during this experiment.

### 2.1 Ferrihydrite Synthesis

Two-line ferrihydrite was synthesized by titrating a 104 mM aqueous solution of ferric chloride hexa-hydrate (Fe^III^Cl_3_·6H_2_O) to a pH of 7.2–7.5 using 1 M sodium hydroxide (NaOH).^32,33^ After hydrolysis, the precipitates were centrifuged and washed thoroughly (5–7 times) with deionized water to remove any traces of salts and then freeze-dried. The freeze-dried powder was stored in an airtight amber glass tube at 4 °C until further use (not longer than a week). Phase purity was confirmed with X-ray diffraction (XRD) analysis before using the ferrihydrite in the experiments (Figure S1).

### 2.2 Sulfide Solution Preparation

A stock solution of (0.5 M) dissolved sulfide was freshly prepared by dissolving sodium sulfide nonahydrate (Na_2_S·9H_2_O) crystals (Acros, Belgium) in O_2_-free Milli-Q inside the anoxic chamber.

### 2.3 Arsenic Sorption and Sulfidation Reaction

Ferrihydrite (240 mg) was added to 120 mL of O_2_-free Milli-Q water (with 0.1 M NaCl as background electrolyte), and 250 μM of either arsenate or arsenite was added to the vials from stock solutions inside an anoxic chamber. Vials were then incubated for 48 h with end-to-end mixing in the dark at ambient temperature to allow As sorption on ferrihydrite nanoparticles. Measured As concentrations in aqueous phase (<0.22 μm) after incubation were <0.7 ppb in all experimental vials, indicating that more than ∼99.8% of the As sorbed to the ferrihydrite surfaces. Dissolved sulfide (from stock solution) was then added to achieve different S/Fe ratios (S/Fe = 0.1–2.0) in the vials except in controls where no dissolve sulfide was added. A set of experimental vials (without As but with sulfide) was also set up with identical S/Fe ratios as a control. After adjusting pH to 5, vials were closed with an airtight septum with aluminum crimps to restrict oxygen penetration and placed on a continuous horizontal shaker (240 rpm). Aqueous and solid-phase samples were taken at different time intervals (30 min to 70 days) inside an anoxic chamber using disposable needles and syringes. The retrieved samples were filtered through 0.22 μm PES filters (Millipore) using a filtration assembly that allows the preservation of the filter paper. The filter paper was allowed to dry under a N_2_ environment inside the anoxic chamber and kept sealed until analyzed using XAS. The aqueous filtrate was immediately distributed to preprepared vials for other analyses. Samples for As speciation in aqueous phase (<0.22 μm) were flash frozen with liquid N_2_ immediately after adding diethylene triamine pentaacetic acid (DPTA) (10 mM) in order to complex any remaining free Fe(II) and kept frozen until analysis and thawed in an anaerobic glovebox before analysis.^26^ Samples for sulfide analysis in aqueous phase (<0.22 μm) were preserved by adding Zn-acetate (10 mM), and samples for total metal analysis were diluted with 2% nitric acid and preserved at 4 °C until analyzed.

### 2.4 Chemical Analysis of Aqueous Solutions

Sulfate and thio-sulfate in aqueous phase (<0.22 μm) were measured in unacidified samples by ion chromatography (IC) using a Dionex DX-100 ion chromatography column. Fe(II) and Fe(III) in aqueous phase (<0.22 μm) were measured using a revised ferrozine method as described by Viollier et al. (2000)^34^ at a wavelength of 562 nm (limit of detection was 0.4 μmol/L) using a Hewlett-Packard Vectra QS 165 spectrophotometer. Sulfide concentration in aqueous phase (<0.22 μm) was measured using the methylene blue colorimetric method.^35^ Total As concentrations were determined by inductively coupled plasma mass spectrometry (ICP-MS), and total Fe and S were measured using optical emission spectrometry (ICP-OES). As species in aqueous phase (<0.22 μm) were separated by IC (Dionex ICS-3000) using an IonPac AS-16/AG-16 4 mm column (gradient program with 2.5–100 mM NaOH at a flow rate of 1.2 mL/min and 50 μL injection volume; without suppressor) and quantified by ICP-MS (XSeries2, Thermo-Fisher).^23^

### 2.5 X-ray Absorption Spectroscopy

Arsenic *K*-edge X-ray absorption near edge structure (XANES) spectra were collected in fluorescence-yield mode at Beamline (BL) 11-2 of the Stanford Synchrotron Radiation Lightsource (SSRL) at SLAC National Accelerator Laboratory, Menlo Park, CA. The dried filter papers were mounted on aluminum holders inside an anoxic chamber and covered with Kapton tape. The aluminum sample holders were then mounted on a cryostat sample rod inside the anoxic chamber, brought to the BL in a liquid N_2_ bath, and were immediately transferred into the liquid He cryostat. To limit the photo-oxidation/reduction of As under the X-ray beam, analyses were performed at *T* < 10 K. The X-ray energy resolution was maintained by a double-crystal Si(111) monochromator, and the energy was calibrated at 11 919 eV, which is the energy position of the first inflection point in the *K*-edge of gold (Au) foil. A minimum of 3–7 spectra were collected for each sample using a 100-element array solid-state Ge detector (Canberra). The Athena software^36^ was used for background subtraction and normalization of XANES spectra. The energy position of As *K*-edge XANES spectra of each sample was then qualitatively compared to those of a large set of experimental As *K*-edge XANES spectra from synthetic model compound^12,28^ in order to evaluate the changes of As oxidation state and chemical form during sulfidation processes.

## 3 Results

### 3.1 Fe Dissolution and Total As Release in Aqueous Phase

**Figure 1:**
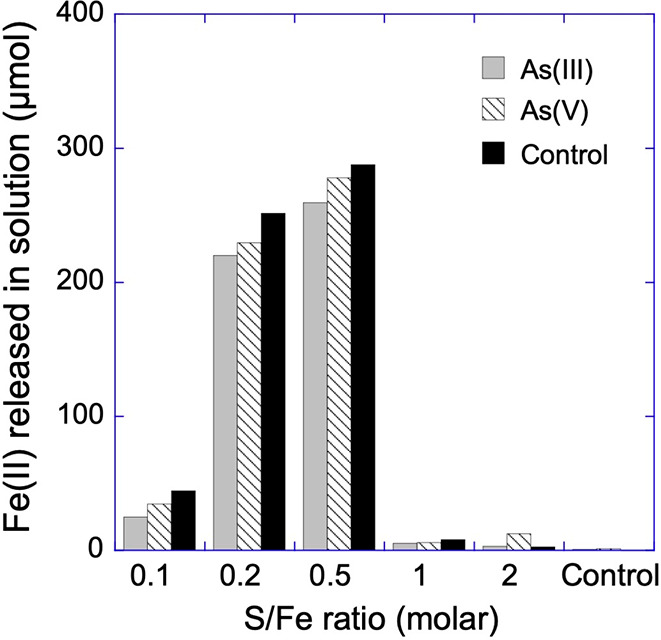
Fe(II) concentrations in aqueous phase (<0.22 μm) measured after 70 days of sulfidation of ferrihydrite at different S/Fe ratios in vials where either arsenite or arsenate was the initial arsenic species added, along with a control where no As was added.

Figure 1 illustrates the release of Fe(II) into the aqueous phase (<0.22 μm) during the reaction of ferrihydrite (with or without surface-sorbed As) with dissolved sulfide at different S/Fe ratios after 70 days. These results suggest that the concentration of Fe(II) released in aqueous phase increased with increasing S/Fe ratio up to 0.5 during the sulfidation of ferrihydrite. However, above the S/Fe ratio of 0.5, the concentration of Fe(II) in aqueous phase dropped significantly in all samples, suggesting the precipitation and aggregation of Fe–S phases.^19,20^ This observation is consistent with previous studies showing similar behavior for ferrihydrite (∼Fe(OH)_3_), goethite (α-FeOOH), and hematite (α-Fe_2_O_3_) during sulfidation at increasing S/Fe ratios.^18^ This behavior was also clearly independent of the presence of sorbed As on the ferrihydrite nanoparticles in our experiments. However, slightly lower Fe(II) concentrations were measured in aqueous phase in the presence of As compared to the control vials.

**Figure 2:**
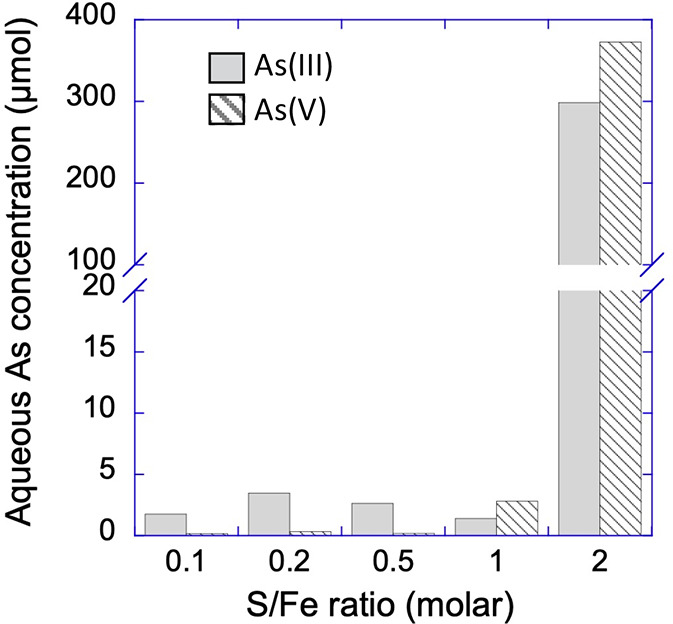
Total arsenic concentrations in aqueous phase (<0.22 μm) measured after 70 days of sulfidation of ferrihydrite at different S/Fe ratios in vials where either arsenite or arsenate was the initial arsenic species added.

Total As concentrations measured by ICP-MS (Figure 2) indicate that significant As release occurred only in the vials with a S/Fe ratio of 2, and As concentrations increased over time independent of the initial As species added (Figure S2). Although the temporal and S/Fe trends were consistent between arsenite and arsenate as initial species, the absolute concentrations released were higher in the arsenate treatments (Figure S2). Dissolved sulfide is expected to be rapidly consumed during reaction with ferrihydrite,^37^ and it was not detected in solution at any sampling point during the experiment (detection limit 5 μM, data not shown)

### 3.2 Aqueous As Speciation Changes at Different S/Fe Ratios

**Figure 3:**
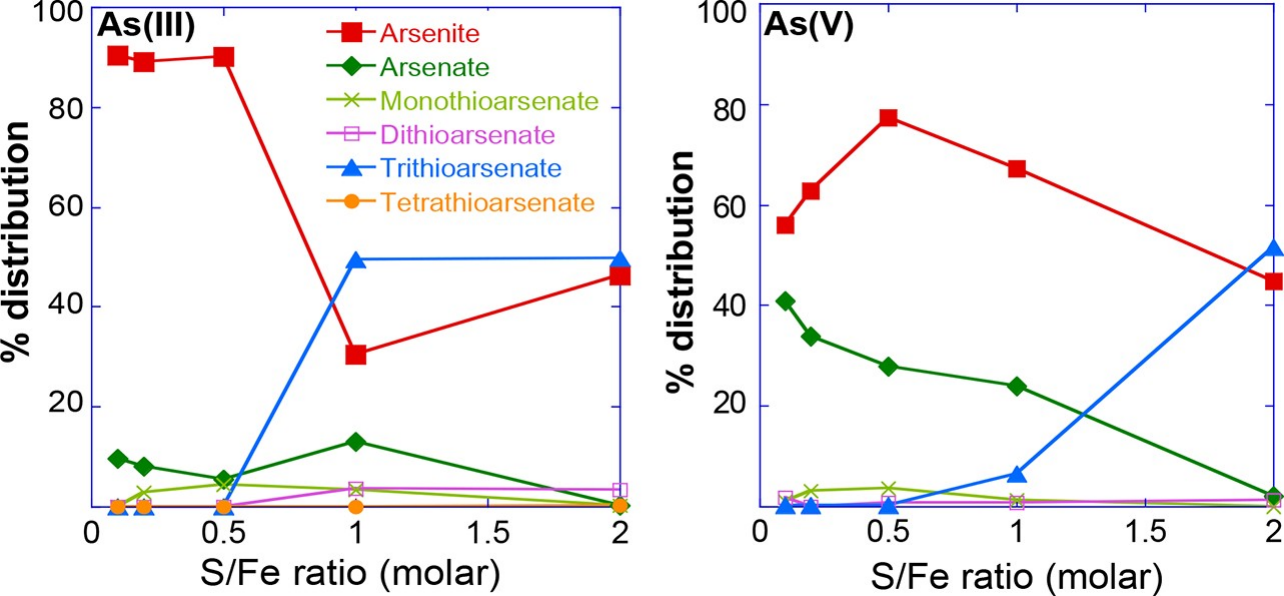
Aqueous arsenic speciation measured after 70 days of reaction at different S/Fe ratios with either arsenite or arsenate as the initial arsenic species in the experiment.

Figure 3 shows the percentage distribution of different As species measured in aqueous phase (<0.22 μm) after 70 days of reaction of dissolved sulfide with arsenite or arsenate sorbed to ferrihydrite at various S/Fe ratios. These results show that, at the end of our experiment (70 days) at a S/Fe ratio of 2, arsenite and trithioarsenate were the two dominant species in the aqueous phase, independent of the initial As species used in our experiments. In both experiments (i.e., with arsenate or arsenite), arsenite and trithioarsenate appear to coexist in equilibrium (∼50 (±5) %) independent of initial As species used in the experiment.

At a S/Fe ratio <2, As-speciation in aqueous phase differs significantly depending on the initial species of As used (Figure S3). It is important to mention here that the As concentrations in aqueous phase at S/Fe < 2 are also significantly lower (Figure 2). In the case of arsenite as the initial species, arsenite concentrations decreased as the dissolved sulfide concentrations (S/Fe ratio) increased in our experiments. Moreover, the arsenite concentration decrease in aqueous phase was directly proportional to the increase in trithioarsenate concentration, suggesting that trithioarsenate forms directly from arsenite without (detectable) intermediate species, which is consistent with previously reported studies.^25^ This is even more evident in the experiment where arsenate was used as the initial As species in the presence of dissolved sulfide. Arsenate was first reduced to arsenite, which then transformed to trithioarsenate, which is also consistent with the mechanism proposed previously.^29,38^

### 3.3 As Speciation Change in Solid Phases at Different S/Fe Ratios

**Figure 4:**
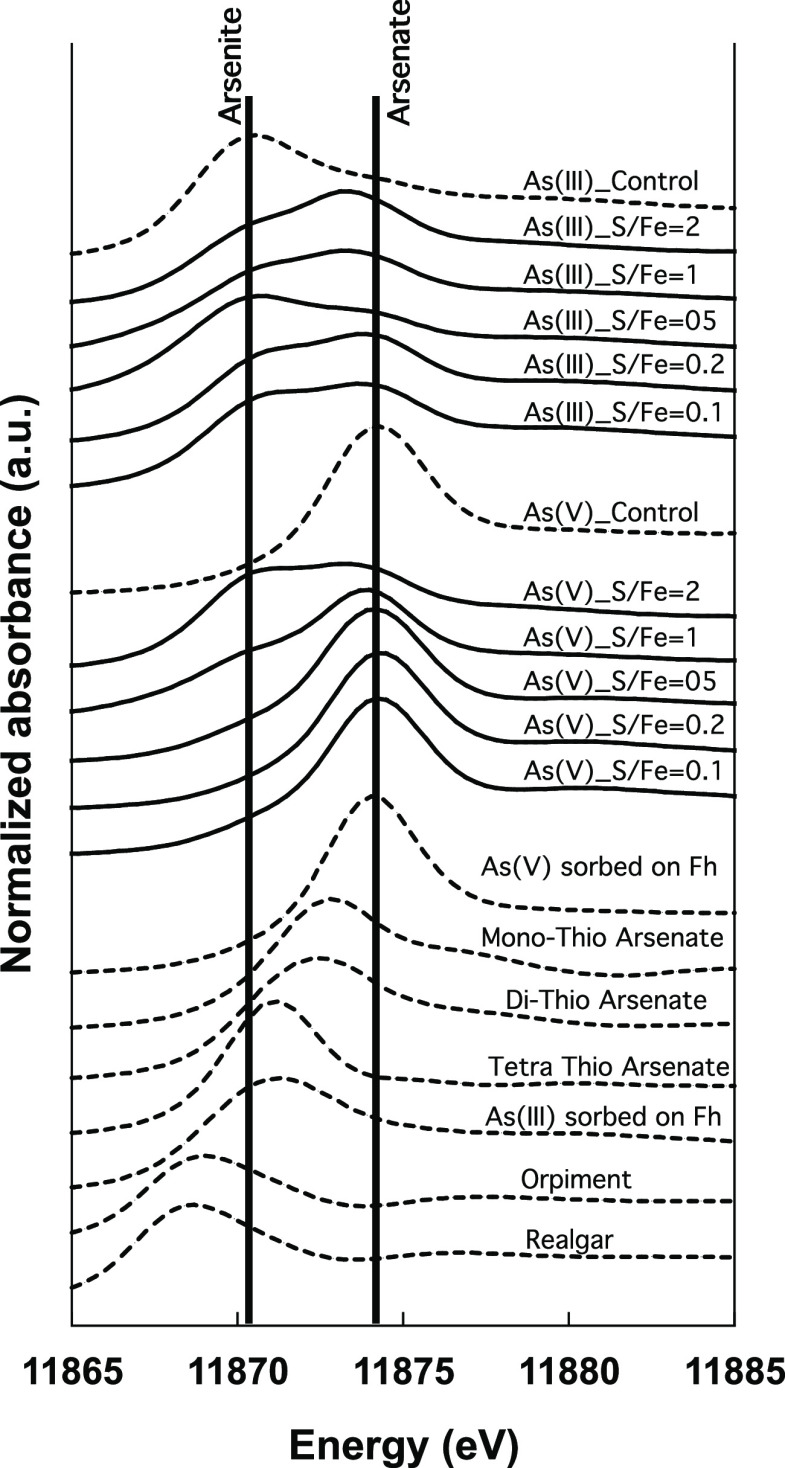
As *K*-edge XANES spectra from the solid phase after 70 days of reaction at different S/Fe ratios batch microcosms using either arsenite or arsenate as the initial arsenic species.

Figure 4 shows the As *K*-edge XANES spectra collected from solid phases in the vials with arsenite or arsenate as the initial As species added. Reference spectra are shown for comparison. In the case of arsenate as the initial sorbed species, results show that most of the As in solid phases remained as arsenate at lower S/Fe ratios (<2); however, at a S/Fe ratio of 2, arsenite and trithioarsenate were the dominant As species. Similarly, in the case of arsenite as the initial sorbed species, As speciation associated with the solid phase also changed with increasing S/Fe ratio. At lower S/Fe ratios (<2) ratios, the presence of arsenate was observed, which is consistent with the speciation of As measured in aqueous phase (<0.22 μm) in these samples (Figure S3). At a S/Fe ratio of 2, the dominant species were arsenite and trithioarsenate, which is similar to the speciation of the aqueous phase observed. The reaction mechanism and speciation changes in the solid phase with different S/Fe ratios appear to be different at lower S/Fe ratios, depending on the initial As species used in the experiment. However, As *K*-edge XANES spectra from the control vials (without sulfide) having surface-sorbed arsenite or arsenate confirmed that As speciation did not change in the absence of dissolved sulfide within the duration of our experiments.

## 4 Discussion

### 4.1 Reductive Dissolution of Ferrihydrite and Fe(II)-S in Aqueous Phase

Fe(II) measured in aqueous phase (<0.22 μm) of our experiments is a product of reductive dissolution of ferrihydrite due to its reaction with dissolved sulfide (reaction 1).
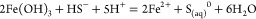
1

Our results support previous observations suggesting that, during reductive dissolution of Fe(III)-(oxyhydr)oxide by dissolved sulfide, it is the sulfide concentration relative to the mass of Fe(III)-(oxyhydr)oxide that controls the reaction mechanism.^18^ It has been previously reported that, at lower S/Fe ratios (≤0.5), Fe(III)-(oxyhydr)oxide dissolution results in the formation of Fe(II)-S complexes and colloids^19^ (mainly nanocluster of FeS)^20^ into aqueous phase, and higher concentrations of sulfide (i.e., S/Fe ratios >0.5) are needed to fully aggregate and precipitate FeS during ferrihydrite sulfidation reactions.^18,19^ The presence of surface-sorbed As (arsenite or arsenate) in our experiment did not change the Fe(II) release behavior; however, the absolute concentrations of Fe(II) were slightly lower in the presence of As, though not significantly (Figure 1). This observation can be explained by noting that, in our experiments, As coverage on ferrihydrite surfaces is low (∼3.15 μM.m^–2^), allowing dissolved sulfide to react with ferrihydrite nanoparticle surface even in the presence of As. Ferrihydrite is poorly crystalline with a small particle diameter (∼5 nm) and high surface area (∼322 m^2^ g^–1^ measured by BET^33^ and theoretical value of ∼530–710 m^2^ g^–1^ as suggested by Hiemstra et al.^39^) and is known to react with dissolved sulfide faster than other common Fe(III)-(oxyhydr)oxides, such as goethite or hematite.^18^

**Figure 5:**
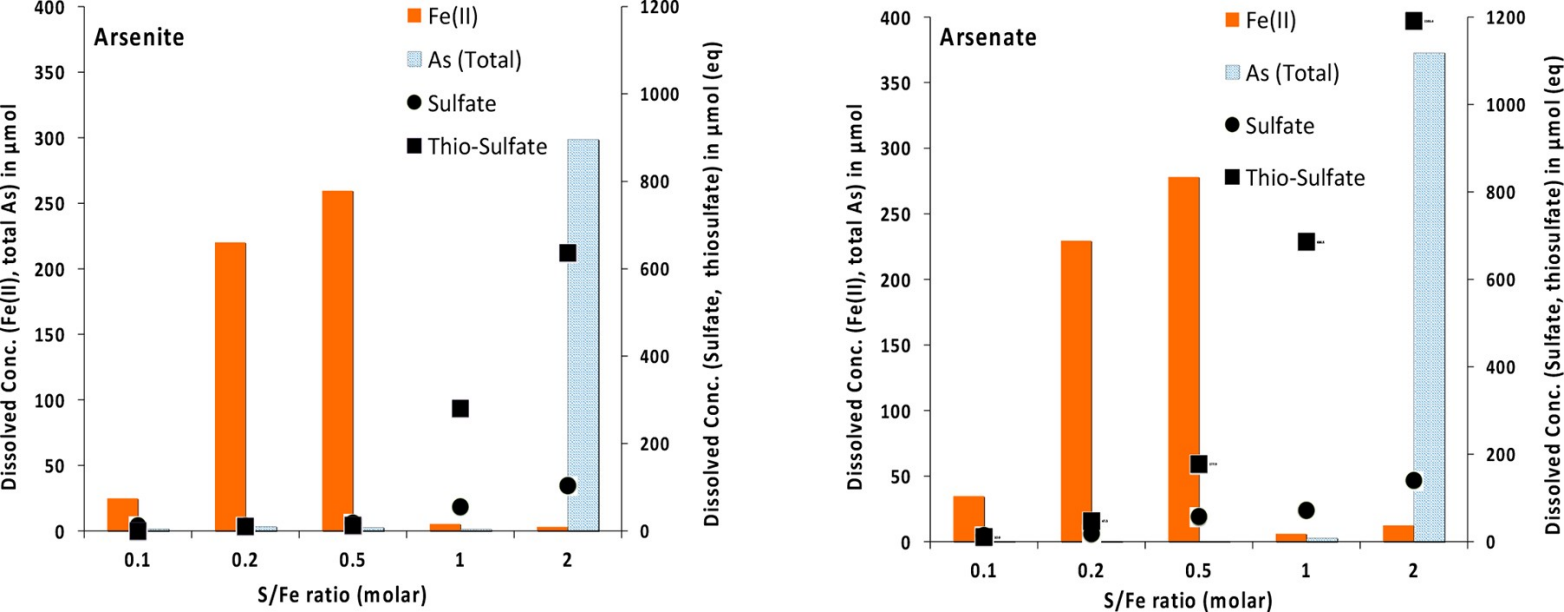
Fe (II), total arsenic, sulfate, and thiosulfate measured in solution at various S/Fe ratios after 70 days of the sulfidation reaction.

Figure 5 shows the pattern of Fe(II) and As release in aqueous phase (<0.22 μm) in our experiments. Although reductive dissolution is an interfacial phenomenon, it did not drive As release into the aqueous phase. In fact, As was released only when there was no more Fe(II) in aqueous phase. This observation suggests that Fe(II) release into aqueous phase is independent of As release and initial speciation and that Fe(II) release precedes As release in our experiments. With As release observed only at a S/Fe ratio of 2 and independent of Fe(II) release, it is likely that As release was driven by a change in mineralogy. X-ray diffraction analysis indicates that, at a S/Fe ratio of 2, ferrihydrite completely transformed to Fe-sulfide.^18^ Arsenate adsorbs primarily to ferrihydrite as a corner-sharing bidentate complex on the apical oxygen of two adjacent edge-sharing Fe-octahedra.^40^ However, typically at lower concentrations, arsenate may also adsorb as a monodentate complex.^40^ Farquhar et al.^41^ observed that arsenite and arsenate sorb on mackinawite surfaces dominantly as inner-sphere complexes binding to one surface sulfide group as monocoordinated species. However, it is well-established that arsenite and thio-As species show a weaker affinity for mackinawite surfaces (and FeS overall) relative to ferrihydrite surfaces,^8,28^ which is consistent with our experimental results. Although arsenate is known to sorb onto FeS relatively strongly, in our experiments, arsenate was reduced to arsenite or transformed to thioarsenate, hence enhancing As mobility in sulfidic environments. Similarly, at low S/Fe ratios (≤0.5), As stays preferentially sorbed to ferrihydrite surfaces because Fe(II) is mainly released as FeS nanocluster in the aqueous phase.^19^ However, Noël et al.^20^ suggested that ferrihydrite sulfidation processes release colloids of ferrihydrite associated with FeS nanoclusters bound to their surface. The remobilization of ferrihydrite colloids could thus promote the mobilization of As, which would explain the small amount of As remobilized at low S/Fe ratios (≤0.5; Figure 2).

### 4.2 Arsenic Speciation Changes and Thio-Arsenic Formation

Thermodynamic predictions suggest that the reduction of Fe(III) to Fe(II) can be coupled with the oxidation of arsenite to arsenate. However, our experimental results only showed a minor fraction (5–15%) of arsenite oxidized to arsenate for S/Fe ratios <1 (Figure S3). This finding is consistent with previous observations by Ona-Nguema et al.^42^ that arsenite oxidation in the presence of Fe(II) also requires the presence of O_2_, which was not the case as our experiments were conducted under strictly anoxic environments. In contrast, on the basis of standard state redox conditions, hydrogen sulfide (H_2_S) is capable of reducing arsenate (H_2_AsO_4_^–^ or HAsO_4_^2–^) to arsenite (H_3_AsO_3_) *via*reaction 2.
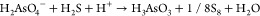
2

This reaction pathway is consistent with our results as we observed significant arsenite concentrations (up to ∼80%) in aqueous medium even at S/Fe ratios <2, when arsenate was the initial As species sorbed on ferrihydrite surfaces (Figure S3), though this transformation was less evident in the solid phase (Figure 4).

The existence of mono-, di-, tri-, and tetra-thioarsenate (Figure S4), in addition to arsenite and arsenate over a wide range of pH conditions (pH 2.1–9.3) have been previously reported in geothermal springs of Yellowstone National Park.^23^ In several natural systems, trithioarsenate has been reported as the dominant aqueous thioarsenic species, for example, in anoxic zones of Mono Lake.^43^ In controlled systems like ours, Planar-Friedrich et al.^25^ have previously shown that trithioarsenite was the primary reaction product of an arsenite solution with excess sulfide, in which case trithioarsenite readily oxidizes abiotically to mainly di- and trithioarsenate (reactions 3–5).
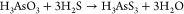
3
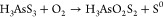
4
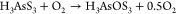
5

Another possible pathway of thioarsenate formation involves the oxidation of arsenite by S^0^ (reaction 6).
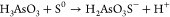
6

Importantly, S^0^ is known to be the dominant oxidation product of sulfide during Fe(III)-(oxyhydr)oxide sulfidation, as shown previously.^18^ Therefore, it can be reasonably assumed that ample S^0^ would have been present in the vials to enable reaction 6 to proceed. This is also consistent with Couture and Van Cappellen’s^44^ assumption that thioarsenates form through the oxidation of thioarsenite by S^0^, as is evident in our experiment where arsenate was reduced to arsenite prior to thioarsenate formation. This is also consistent with the procedures used to prepare thioarsenate salts in the laboratory.^45^ However, Besold et al.^29^ and Planar-Friedrich et al.^38^ have previously shown that S^0^ can only contribute to the formation of monothioarsenate and, for higher thioarsenate formation, an excess of sulfide would be needed.

Under sulfidic conditions, there is also a thermodynamic possibility that As-sulfide phases will precipitate when As and dissolved sulfide concentrations are high in the aqueous phase; however, we did not observe As-sulfide precipitation in our experiments on the basis of As *K*-edge XANES analysis, though we did observe the formation of Fe(II)-sulfide phases. At the S/Fe ratios of 2, ferrihydrite converted completely to Fe-sulfide phases as shown previously by Kumar et al.^18^ by XRD analysis.

In our experiments, due to an excess of dissolved sulfide at S/Fe ratio 2 (S/As ratio ∼22) (Figure S5), the formation of trithioarsenate is likely to be controlled by the initial formation of thioarsenite and finally trithioarsenate, which dominated in solution (reactions 5 and 6). Also, at a S/Fe ratio of 2, this transformation is independent of the initial arsenic species used in the experiment, as in both cases after 70 days of reaction the end products were arsenite and trithioarsenate. However, arsenate was likely reduced to arsenite before forming trithioarsenate in our experiments, suggesting that trithioarsenate formed from arsenite and not directly from arsenate.^29,38^

It is well-established that thiolated-As species exhibit different sorption behavior and affinity than arsenite and arsenate species in the presence of Fe-oxides and Fe-sulfides.^21,30^ Arsenite and thioarsenic species show a weaker affinity for mackinawite than for ferrihydrite, suggesting that the transformation of Fe(III)-(oxyhydr)oxides to FeS can increase As mobility.^13,30^ The concurrent formation of FeS has been previously related to thioarsenate formation and As mobilization.^46^ This explains the release of As in our experiments at higher sulfide concentrations. In our experiment, at a S/Fe ratio of 2, all the ferrihydrite was transformed to FeS as observed by XRD analysis (Figure S1). One reason for the change in sorption affinity of arsenic between ferrihydrite and mackinawite surfaces is likely to be the pH_PZC_ values. Ferrihydrite exhibits a pH_PZC_ value of 7.9,^31^ whereas the pH_PZC_ values for mackinawite and pyrite are 2.9 and 2.4, respectively.^47^ The lower pH_PZC_ value for Fe-sulfides implies that their surfaces will be highly negatively charged, resulting in electrostatic repulsions at our experimental pH values.

It is important to mention here that, once we set the initial pH (after the addition of dissolved sulfides) in the vials, we did not try to control pH in our experiments. In our experiment at S/Fe ratios <1, pH did not change significantly during the experimental period; however, at S/Fe ratios >1, pH increased to 8.5 in control experiments (without arsenic), but the pH increase was significantly lower in the presence of As (i.e., pH values increased up to 6.3 and 6.7 for arsenate and arsenite, respectively (Table S1), perhaps owing to proton release *via*reaction 6). This would perhaps also reflect in the mineral transformation of ferrihydrite in the presence and absence of As, which was not considered in this study. This last point underlines the need to investigate in the future the potential impact of the presence of As on the mechanisms of sulfidation of Fe(III)-(oxyhydr)oxides.

## 5 Environmental Implications

This study has shown that As behavior in reduced sulfidic environments is controlled by the S/Fe ratio available, and the codiagenesis of Fe–As–S species determines the environmental fate of As in these systems. Our results challenge the conventional wisdom that sulfate reduction can mobilize or immobilize arsenic. In addition, they show, perhaps for the first time in greater detail, the complexity and the partitioning of speciation of As–Fe–S in these systems driven by dissolved sulfide concentrations. Also, with the increasing recognition of the environmental relevance and importance of thiolated arsenic species, our results provide much-needed information on the mechanism of formation, partitioning, mobility, and adsorption behavior in reduced environmental systems by elucidating the decoupling of As and Fe codiagenesis in sulfidic environments. These processes could potentially have serious implications, for example, for climate change-driven higher sea-level rise and submergence of coastal areas and riverbeds or seasonal water table increases driving redox heterogeneity (sulfidation vs oxidation) and consequent contaminant mobility and water quality. However, our current study was conducted using controlled lab experiments. Further studies with variable environmental conditions are underway, but this gives a first mechanistic understanding of system behavior and predictability.

## Supporting Information Available

Figures of X-ray diffraction data, aqueous arsenic speciation data, chemical speciation and structures of thioarsenate complexes, and final S/Fe vs S/As ratio in our experiment and table of pH values measured in all the experimental vials after 70 days (PDF)
