# Supplementary material for: Mechanism of Arsenic Partitioning During Sulfidation of As-Sorbed Ferrihydrite Nanoparticles
Source: ACS Earth Space Chem. 2022 Jul 6;6(7):1666–73. doi: 10.1021/acsearthspacechem.1c00373 (PMC9310089; doi:10.1021/acsearthspacechem.1c00373)
Supplement: Supplementary file 1 — sp1c00373_si_001.pdf [file sp1c00373_si_001.pdf]

Supporting Information

For

Mechanism of Arsenic Partitioning During Sulfidation of As-sorbed Ferrihydrite Nanoparticles

Naresh Kumar[1,2,3*], Vincent Noël[4], Johannes Besold[5], Britta Planer-Friedrich[5], Kristin Boye[4], Scott Fendorf [6], and Gordon E. Brown Jr.[1,2,4]

[1]Department of Geological Sciences, School of Earth, Energy & Environmental Sciences, Stanford University, Stanford, CA 94305-2115, USA
[2]Center for Environmental Implications of NanoTechnology (CEINT), Duke University, Durham, NC 27708, USA
[3] Department of Environmental Sciences, Soil Chemistry and Chemical Soil Quality Group, Wageningen University, 6708 PB Wageningen, The Netherlands.
[4]Stanford Synchrotron Radiation Lightsource (SSRL), SLAC National Accelerator Laboratory, 2575 Sand Hill Road, Menlo Park, CA 94025, USA
[5]Environmental Geochemistry, Bayreuth Center for Ecology and Environmental Research (BayCEER), University of Bayreuth, D-95440 Bayreuth, Germany.
[6]Department of Earth System Sciences, School of Earth, Energy & Environmental Sciences, Stanford University, Stanford, CA 94305, USA

*Corresponding author: naresh.kumar@wur.nl
ORCiD : 0000-0002-8593-5758

Department of Environmental Sciences
Soil Chemistry and Soil Quality Group
Wageningen University
Droevendaalsesteeg 3A, 6708 PB
Wageningen, The Netherlands

Content:

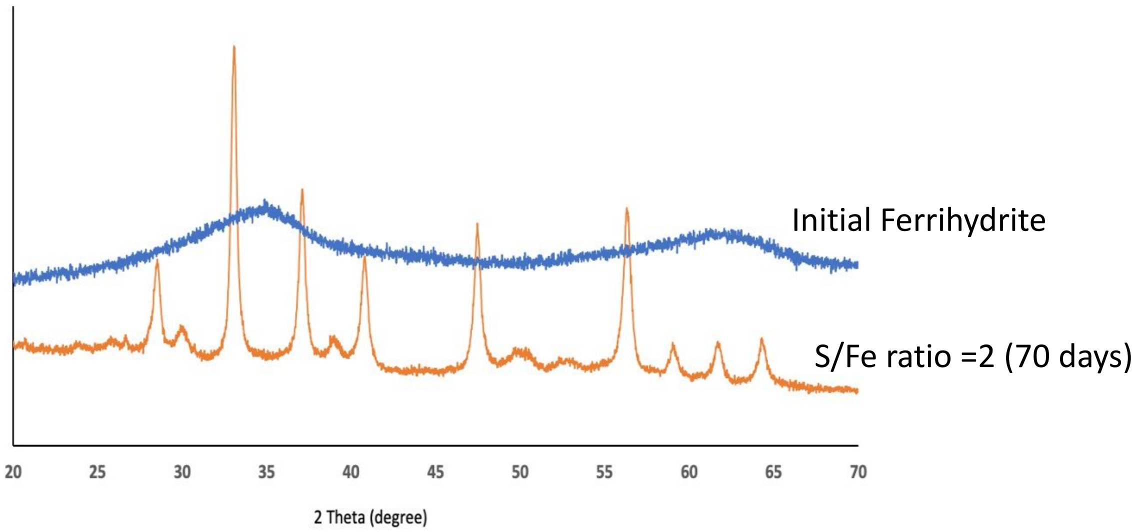

Figure S1: XRD diffractogram of 2-line Ferrihydrite used in this study and the reacted product at 70 days at S/Fe=2

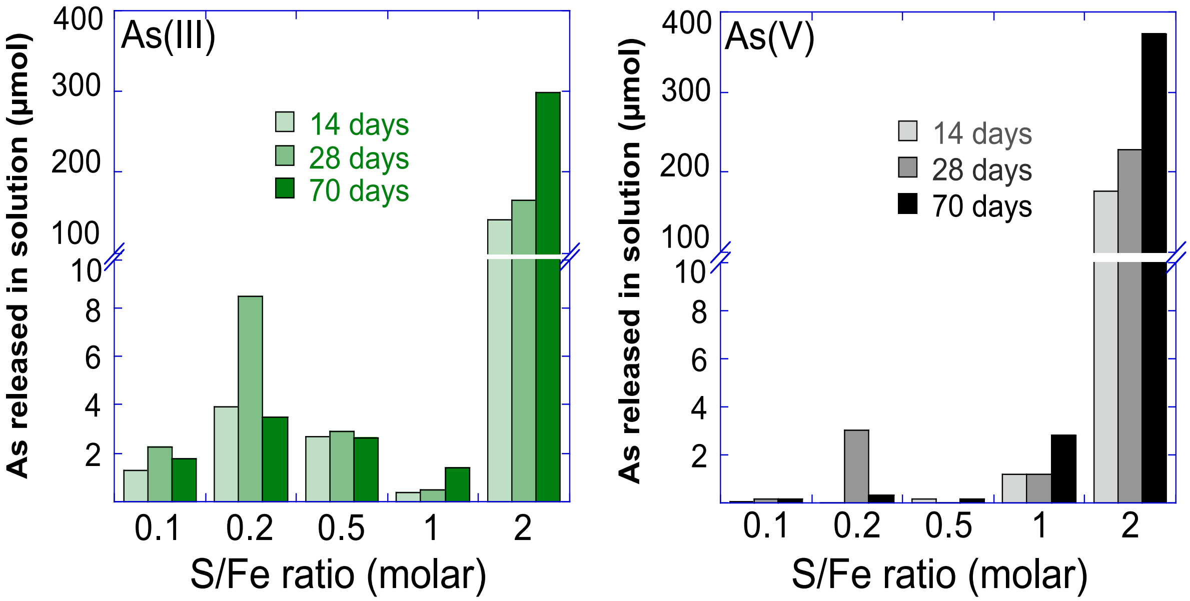

Figure S2: Total dissolved arsenic concentrations at different time points during the experiment for arsenite and arsenate experiments

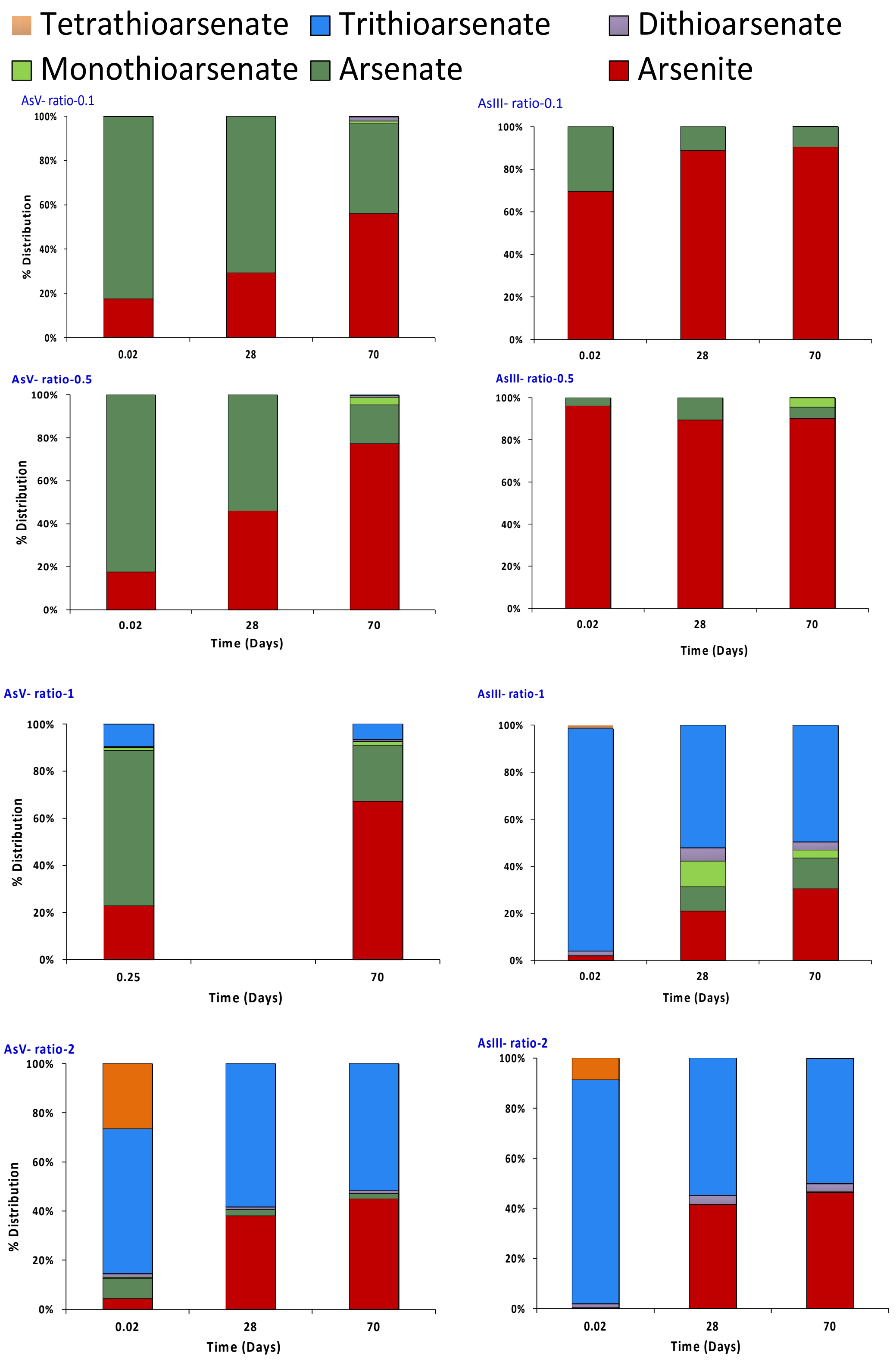

Figure S3: **Aqueous Arsenic speciation during sulfidation reaction of ferrihydrite and dissolved sulfide at various S/Fe ratios for experiments using arsenate (As(V)) and arsenite (AS(III)) as initial arsenic species.**

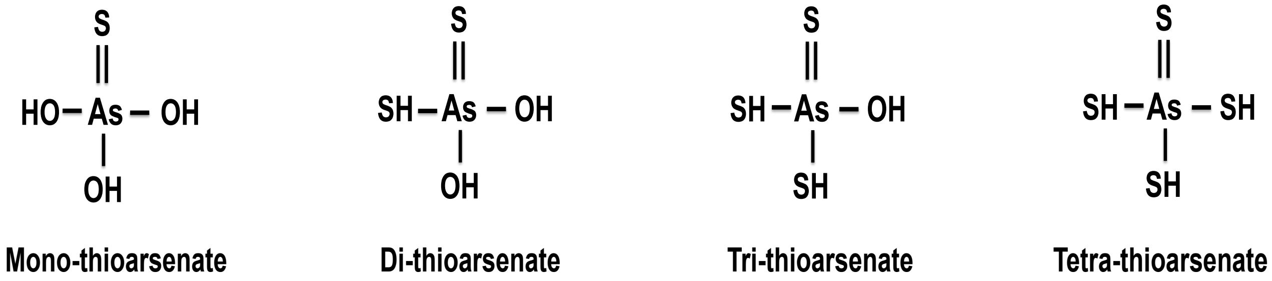

Figure S4: **Chemical speciation and structures of thioarsenate complexes.**

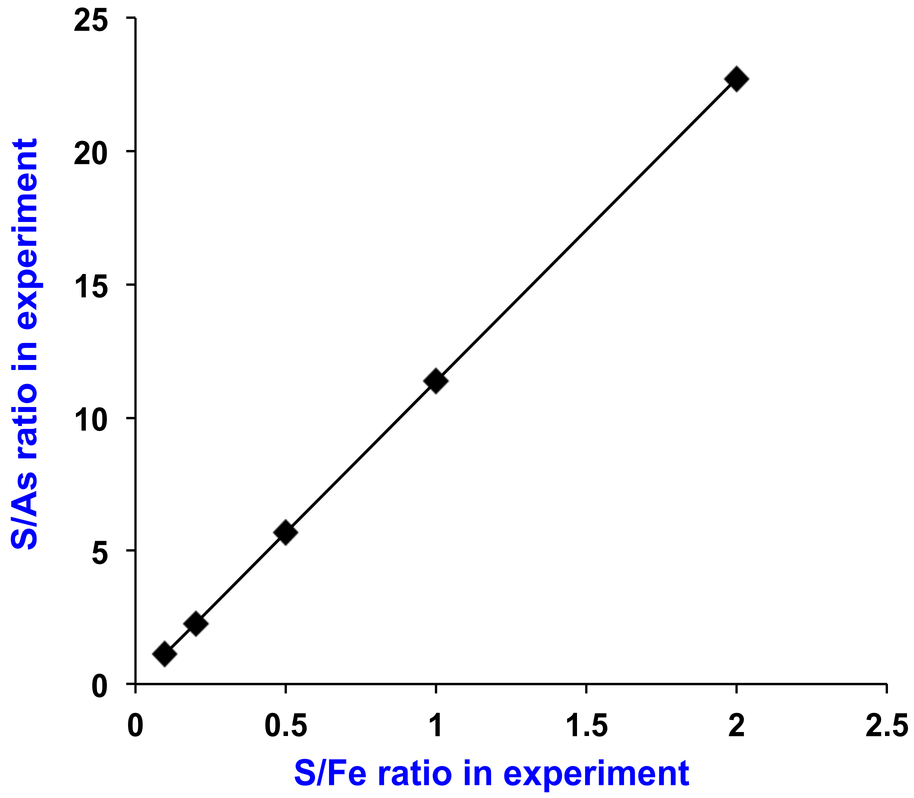

Figure S5: **Final S/Fe *vs* S/As ratio in our experiment.**

Table S1: **pH values measured in all the experimental vials after 70 days**

	Treatment	Ratio	pH
1	Control (Ferrihydrite+ Sulfide)	Control	
2		0.1	4.61
3		0.2	4.15
4		0.5	3.75
5		1	8.22
6		2	8.52
7	Ferrihydrite+As(V)+ Sulfide		
8		Control	5.02
9		0.1	4.51
10		0.2	4.33
11		0.5	4.74
12		1	6.63
13		2	6.28
14	Ferrihydrite+As(III)+ Sulfide		
15		Control	5.11
16		0.1	4.51
17		0.2	4.11
18		0.5	4.92
19		1	6.45
20		2	6.74
